# A ViTUNeT-based model using YOLOv8 for efficient LVNC diagnosis and automatic cleaning of dataset

**DOI:** 10.1515/jib-2024-0048

**Published:** 2025-06-04

**Authors:** Salvador de Haro, Gregorio Bernabé, José Manuel García, Pilar González-Férez

**Affiliations:** Computer Engineering Department, 16751 University of Murcia, 30100 Murcia, Spain

**Keywords:** data analysis, image detection, left ventricular non-compaction diagnosis, medical imaging, convolutional neural networks

## Abstract

Left ventricular non-compaction is a cardiac condition marked by excessive trabeculae in the left ventricle’s inner wall. Although various methods exist to measure these structures, the medical community still lacks consensus on the best approach. Previously, we developed DL-LVTQ, a tool based on a UNet neural network, to quantify trabeculae in this region. In this study, we expand the dataset to include new patients with Titin cardiomyopathy and healthy individuals with fewer trabeculae, requiring retraining of our models to enhance predictions. We also propose ViTUNeT, a neural network architecture combining U-Net and Vision Transformers to segment the left ventricle more accurately. Additionally, we train a YOLOv8 model to detect the ventricle and integrate it with ViTUNeT model to focus on the region of interest. Results from ViTUNet and YOLOv8 are similar to DL-LVTQ, suggesting dataset quality limits further accuracy improvements. To test this, we analyze MRI images and develop a method using two YOLOv8 models to identify and remove problematic images, leading to better results. Combining YOLOv8 with deep learning networks offers a promising approach for improving cardiac image analysis and segmentation.

## 1 Introduction

Cardiovascular diseases represent a major global health issue, contributing to a large share of mortality worldwide [1], 2]. Among these, *Left Ventricular Non-Compaction* (LVNC) is a distinctive form of cardiomyopathy. Abnormal trabeculations mark LVNC [3] within the *left ventricle* (LV) and is often linked to other types of cardiomyopathies [4], [5], [6]. Despite several proposed methods using *magnetic resonance imaging* (MRI) for quantifying left ventricular trabeculae, there remains no consensus on a universal standard, resulting in manual and subjective diagnosis methods that can be time-consuming [7], [8], [9], [10], [11]. A common approach involves calculating the *trabecular volume* (TV) as a percentage of the total volume of the non-compacted left ventricular wall.

In recent years, medical image segmentation has progressed significantly, particularly in radiology and diagnostic imaging, mainly due to advancements in machine learning and *convolutional neural networks* (CNNs). In 2022, we introduced the *Deep Learning for Left Ventricular Trabecular Quantification* (DL-LVTQ) [12] tool, which was a significant step forward. Using the UNet architecture [13], this tool allowed for rapid and precise segmentation of medical images, providing potential for LVNC diagnosis by effectively identifying the endocardial border and trabeculae.

Later improvements and adaptations of DL-LVTQ [14], 15] further expanded its potential, promising to diagnose LVNC across different patient populations with various cardiomyopathies. One of the most significant advancements in medical image segmentation based on U-Net has been the development of nnU-Net, an automated and self-adapting framework for U-Net-based segmentation, which has achieved state-of-the-art performance across multiple biomedical imaging tasks [16].

Although CNN-based methods have achieved considerable success, they face challenges in modeling long-range dependencies between image elements due to the limited size of the convolution kernel. To address this, the Transformer architecture, initially developed for sequence-to-sequence tasks, has been applied in medical image segmentation, resulting in models like Attention U-Net and its variants. While Transformers excel at capturing global context, they struggle with fine-grained local details. To overcome this limitation, researchers have combined CNNs with Transformer architectures [17], as demonstrated in models like TransUNet [18], which merges local feature extraction with global context modeling via self-attention.

Building on the work of Pandey et al. [19], who combined YOLOv8 [20] and SAM [21] models for *region of interest* (ROI) segmentation in medical applications, we have recently proposed the following objectives of a work [22].

– Train a YOLOv8 model to detect the left ventricle in MRI slices, guiding convolutional models to focus segmentation on the ROI.
– Investigate the use of attention mechanisms and Transformer-based architectures for LVNC diagnosis through a model that integrates the strengths of U-Net and Transformer networks.
– Evaluate segmentation model performance using YOLOv8 preprocessing, applying relevant metrics.

Therefore, and building on the above-mentioned previous work and foundations, our study has the following objectives:

– Analyze the current dataset to identify MRI slice types that limit performance improvements.
– Develop a method that automatically identifies problematic MRI slices.

## 2 Materials and methods

### 2.1 Hyper-trabeculation quantification in the left ventricule

Following the approach of previous works [12], 14], 15], 22], the metric we are interested in calculating is the percentage of TV relative to the total volume of the ventricular wall. This value is easily computed using the following formula:

$$TV\%=100\cdot \frac{Trab.volume}{Trab.volume+Compac.Volume}\%$$

where:

– **Trabecular volume (Trab. volume):** Refers to the total volume occupied by the trabeculated myocardium in the left ventricle.
– **Compact myocardial volume (Compac. volume):** Represents the volume of the compacted myocardium, excluding trabeculated structures.

A high TV% is recognized in the literature as a marker for LVNC. Consistent with previous research [14], we applied a validated threshold of 27.4 %.

**Figure 1:**
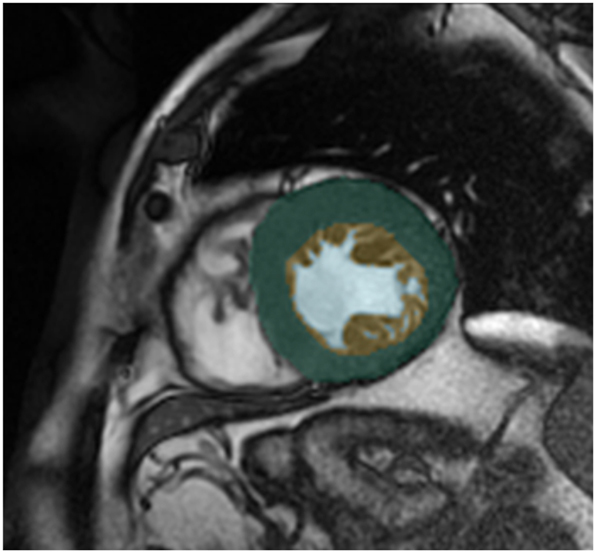
Segmentation of the left ventricle, illustrating the external layer (green), the inner cavity (blue), and the trabeculae or non-compacted area (yellow).

The volumes in the above formula are approximated as the sum of areas across all MRI slices of a patient. The areas of a specific slice are calculated using the segmentation mask obtained by a deep learning model, as shown in Figure 1.

### 2.2 Current configuration of the cardiology dataset for LVNC

Continuing with the expansion work of the initial dataset (which we will refer to as the “old dataset” throughout the article), which we began in our previous study [22], we strengthen the dataset by incorporating new images. These magnetic resonance imaging (MRI) scans come from multiple hospitals and were provided by cardiologists from those institutions. All images have been fully anonymized to comply with data protection regulations, ensuring patient confidentiality. The dataset now consists of 3,459 short-axis MRI slices from 450 patients, categorized into several groups:

– **P** includes 293 patients with *hypertrophic cardiomyopathy* (HCM) [23].
– **H** contains 78 patients (previously 28) diagnosed with LVNC based on Petersen et al.’s criteria [24], which define LVNC as a non-compacted to compacted (NC/C) *ratio* > 2.3 in end-diastole, measured in short-axis MRI images.
– **X** consists of 69 patients (previously 58) with various cardiomyopathies that cannot be distinctly classified, ranging from non-compaction cardiomyopathy to arrhythmogenic cardiomyopathies of the *right ventricle* (RV) or LV, dilated cardiomyopathy, HCM, unclassifiable or mixed cardiomyopathies, and hereditary cardiomyopathies.
– **T** includes 10 patients with titin-related cardiomyopathy [25], a key protein necessary for normal cardiac function, where mutations can cause structural abnormalities in the heart muscle.

**Figure 2:**
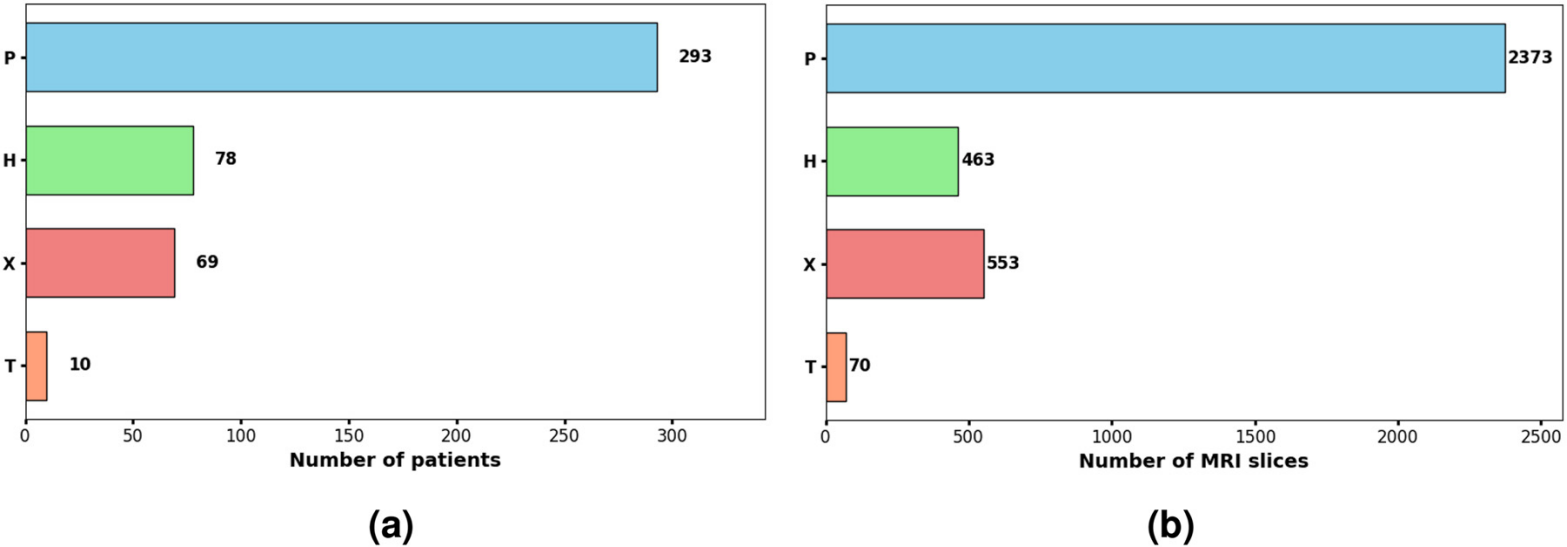
Comparison between the number of patients and MRI slices in each group. (a) Numbers of patients in each group. (b) Numbers of MRI slices in each group.

Figure 2 graphically illustrates the current configuration of the dataset.

It is important to highlight that, for this study, many of the newly added patients are healthy individuals with minimal trabeculations. This is because most of the available MRI slices present a ring of trabecular tissue around the internal cavity of the left ventricle, something common in the MRI slices of patients from group P, which accounts for around 70 % of the dataset. As a result, segmentation models tend to learn this pattern and can hallucinate when encountering slices with few trabeculations that do not follow the typical pattern.

It is also worth mentioning that the number of available slices per patient varies depending on the acquisition protocol and the field of view of the MRI scan. However, on average, each patient has approximately 7 available slices, corresponding to short-axis slices acquired during the diastolic phase.

### 2.3 YOLOv8 models used

We utilized the Python library developed by Ultralytics [20] to create, train, and evaluate our YOLOv8 models due to its efficiency and seamless integration with PyTorch.

In our experiments, we selected YOLOv8m to balance accuracy and computational efficiency. Smaller variants (YOLOv8n, YOLOv8s) require fewer resources but underperform in medical imaging tasks, while larger versions (YOLOv8l, YOLOv8x) significantly increase computational demands without substantial accuracy gains. Thus, YOLOv8m was chosen for its optimal trade-off between performance and feasibility.

#### 2.3.1 YOLOV8 for LV detection

Training a YOLO model for object detection involves labeling the images in a defined format. These labels are saved in accompanying text files, with each line corresponding to an object in the image. The format used is as follows:

[object−class][x][y][width][heigth]

Object-class is an integer representing the object class; x is *x* coordinate of the bounding box center, normalized to image width; y is *y* coordinate of the bounding box center, normalized to image height; width is the width of the bounding box, normalized to image width and, finally, height is the height of the bounding box, normalized to image height.

**Figure 3:**
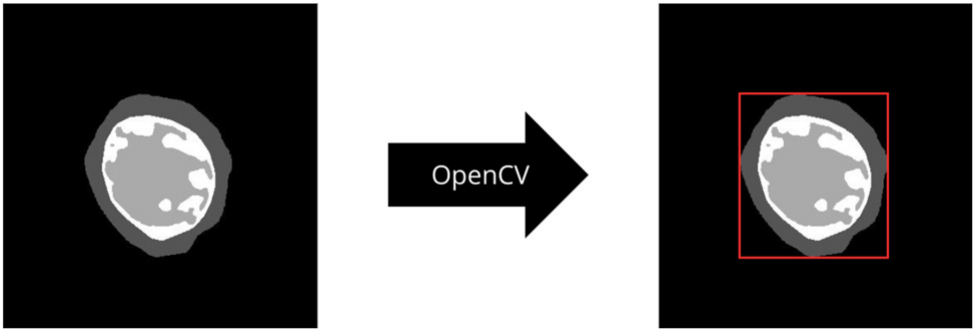
OpenCV library is used to find the outermost contour in segmentation masks.

To streamline the labeling process, we make use of segmentation masks (ground truth) provided for our MRI slices. For each slice, we first identify the largest contour in the segmentation mask, which outlines the outer boundary of LV. Based on this contour, we then create a bounding box that encapsulates the entire left ventricle complex. The OpenCV library offers functions to detect the largest contour in segmentation masks (see Figure 3).

To train the YOLOv8 model, the dataset was randomly split into 80 % for training and 20 % for validation, without grouping images by patient. Since YOLOv8 functions as a region-based detector, enforcing a per-patient split was initially considered unnecessary for this task. However, we acknowledge that multiple slices from the same patient may share morphological similarities, which could lead to an optimistic bias in the evaluation results. We include this as a potential limitation of our approach and plan to explore patient-level splitting in future work to assess its impact on generalization. In addition, no stratification by class (i.e., presence or absence of LVNC) was applied during the split, which could result in class imbalance between training and validation sets. We will consider applying stratified sampling in future studies to ensure more balanced distributions across data splits.

**Figure 4:**
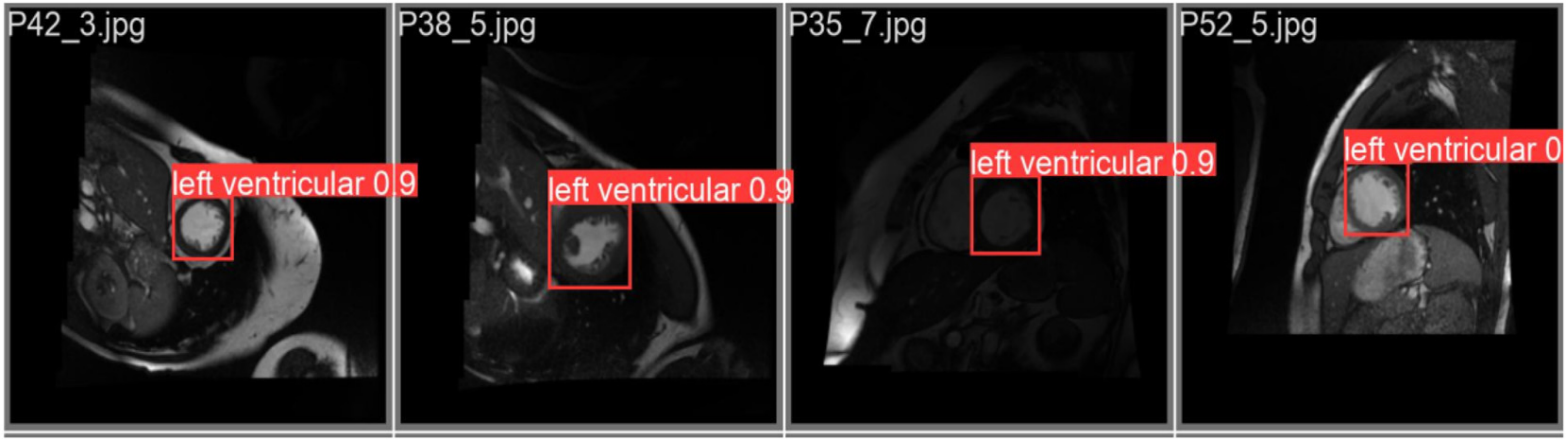
Performance of the YOLOv8m model trained on the cardiology dataset.

After training, the model is assessed on the test set, achieving an mAP50-95 score of 0.928. This score reflects high accuracy in detecting the left ventricle in short-axis MRI images (see Figure 4).

#### 2.3.2 Dataset cleaning with YOLOv8 models

We suspect that the segmentation models’ accuracy in this study may be limited by the quality of the cardiology dataset we are using.

Our research group, in collaboration with cardiologists from Hospitals Virgen of Arrixaca and Vall d’Hbron in Murcia and Barcelona (Spain), is developing an artificial intelligence-based application that enables the automatic diagnosis of LVNC. A complete magnetic resonance study typically consists of a series of slices, and each slice contains a series of frames. For the diagnosis of LVNC, among other things, cardiologists are particularly interested in the frames corresponding to the tele-diastolic phase (most expanded LV) and the tele-systolic phase (least expanded LV). To avoid specialists from using other applications or relying on their visual judgment to determine these phases, we have trained and introduced a YOLOv8 model into our application to detect the inner cavity of LV, allowing us to measure the area of the bounding box generated by the model. The tele-diastolic phase will have the largest area, and the tele-systolic phase the smallest.

**Figure 5:**
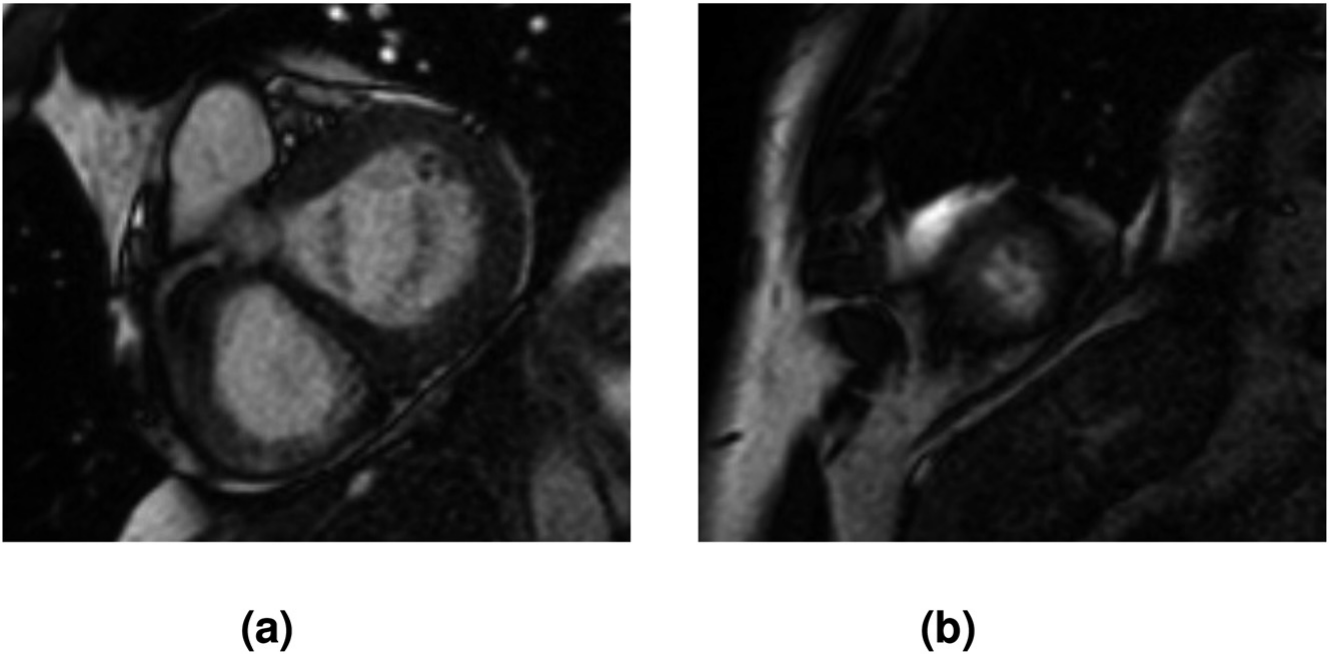
Examples of slices that cause issues for the models. (a) MRI slice no ring. (b) MRI slice showing LV with small area and artefacts.

In this line of work, over the past few months and in collaboration with several specialists, we have concluded that certain types of MRI slices are entirely discardable when diagnosing LVNC and may be affecting the training quality of convolutional models. We will classify these slices into the following categories:

– **No Ring**: This category includes MRI slices where the external layer of the LV does not form a closed ring around the inner cavity. We can find an example of this type in Figure 5a.
– **Poor Quality:** This category includes MRI slices with a small area, typically basal or extreme, with numerous artifacts and poor-quality LV. A slice from this category is shown in Figure 5b.

**Figure 6:**
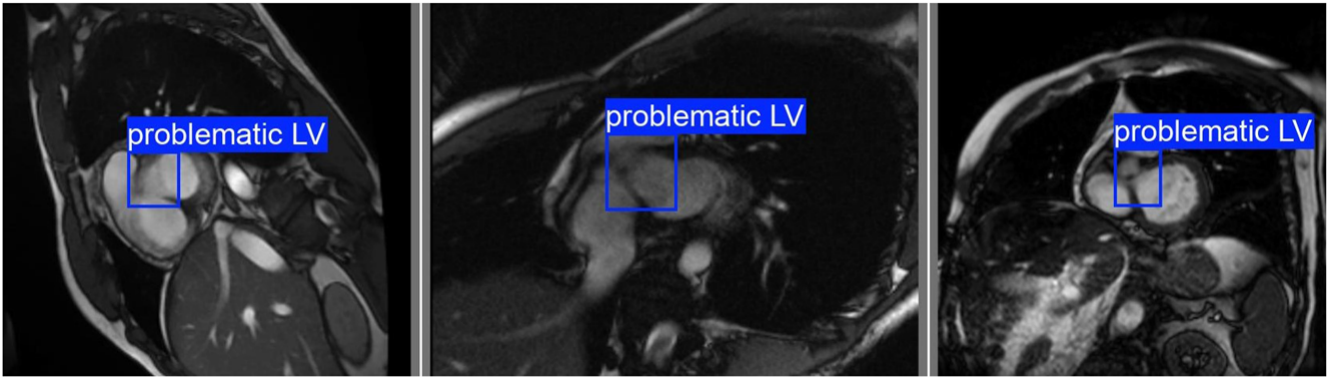
Detections made by model opening-IC-LV on slices of the No Ring class.

Our goal is to develop a method for automatically detecting MRI slices related to these two classes. To achieve this, we trained two YOLOv8 models:

– **Model Opening-IC-LV**: We utilize this model to detect openings in the outer layer surrounding the internal cavity of the left ventricle. To train this model, approximately 400 images of the No Ring class were collected and labeled. The detections made by this model can be seen in Figure 6.
– **Model High-Quality-IC-LV**: The purpose of this model is to detect a high-quality internal cavity in the left ventricle. With this model, we can determine when we are observing a well-resolved internal cavity and measure its area using a bounding box. This model is trained using a process similar to the one described in the previous section, utilizing segmentation masks.

Thanks to the Ultralytics library [20], when we apply a YOLO model to an image, we obtain an object with several attributes, including the confidence with which the model believes it has detected what it was trained for. We can also obtain the central coordinates, as well as the width and height of the generated bounding box. In this way, with Model Opening-IC-LV and Model High-Quality-IC-LV trained for their respective purposes, we process the new cardiology dataset with both models and obtain, for each MRI slice, the following values:

– **Conf-Valid-IC**: Confidence with which Model High-Quality-IC-LV believes it has detected a valid internal cavity.
– **Conf-Open-IC**: Confidence with which Model Opening-IC-LV believes it has detected an opening in the internal cavity (i.e., confidence that it is a No Ring slice).
– **Area-bbox-Valid-IC**: Area of the bounding box generated by Model High-Quality-IC-LV when detecting the internal cavity.

Thus, an MRI slice is represented by a triplet of values:

(Conf−Valid−IC,Conf−Open−IC,Area−bbox−Valid−IC)

**Figure 7:**
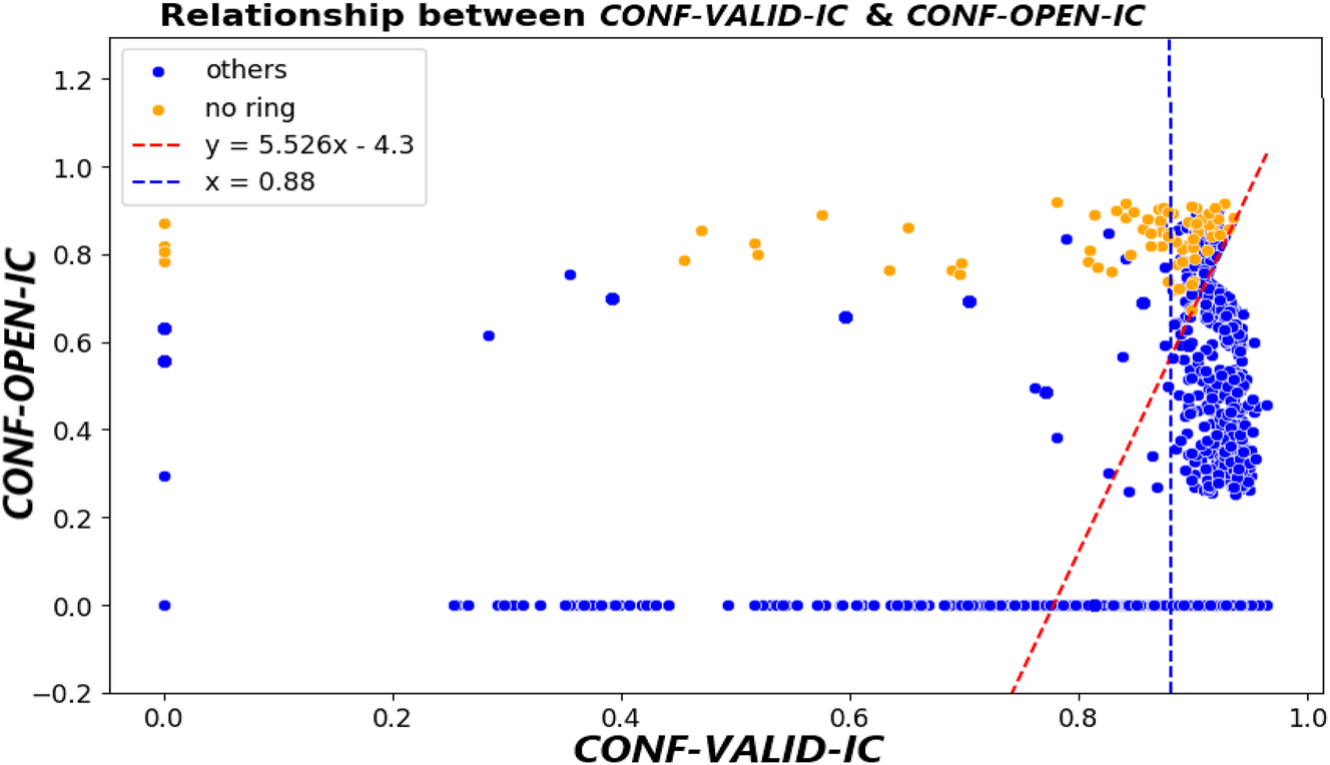
Relationship between *Conf*-*Valid*-*IC* and *Conf*-*Open*-*IC*, and the linear model (red line) that best distinguishes No Ring and poor-quality cuts (low *Conf*-*Valid*-*IC* value) from the rest.

Now, based on these values, our objective is to find a pattern or model capable of determining when an MRI slice is discardable or not. To this end, Figure 7 shows the relationship between values *Conf* − *Valid* − *IC* and *Conf* − *Open* − *IC* for the dataset slices is shown, where the slices manually labeled as No Ring to train model Y are displayed in orange. In this way, Figure 7 shows a linear model that best divides the No Ring slices from the rest of the slices. We also establish a threshold for the confidence of a valid internal cavity, i.e., a threshold for the *Conf* − *Valid* − *IC* values. This confidence threshold is set at 0.88, as we observe that most slices initially classified as No Ring are above this threshold.

In this way, the linear model from Figure 7 provides the following plane separation:

(1) $$\left\{\begin{array}{cc} Y-5.526\times X>-4.3\; & \left(1.1\right)\\ Y-5.526\times X\leq -4.3\; & \left(1.2\right) \end{array}\right.$$

**Figure 8:**
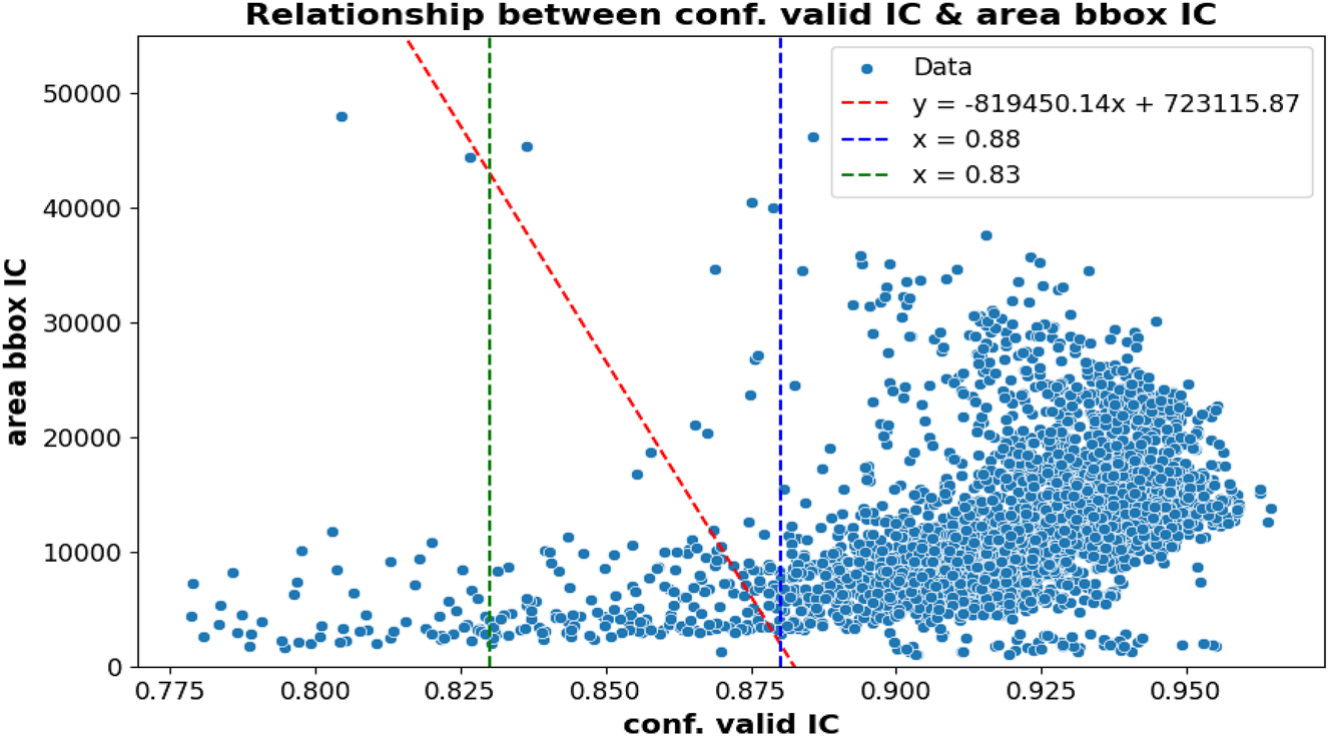
Relationship between values *Conf* − *Valid* − *IC* and *Area* − *bbox* − *Valid* − *IC* of the MRI slices.

The slices (*Conf* − *Valid* − *IC*, *Conf* − *Open* − *IC*, *Area* − *bbox* − *Valid* − *IC*) that satisfy inequality (1.1) are discarded, as they are slices with a high value of *Conf* − *Open* − *IC* (high confidence of being from the No Ring class), others with *Conf* − *Valid* − *IC* = 0 (Model High-Quality-IC-LV does not detect an internal cavity at all), or with *Conf* − *Valid* − *IC* lower than 0.775 (a poor-quality internal cavity). For the slices that satisfy inequality (1.2), we conduct a study on the relationship between values *Conf* − *Valid* − *IC* and *Area* − *bbox* − *Valid* − *IC*, as there are slices with *Conf* − *Valid* − *IC* lower than the 0.88 threshold, and we want to assess whether they are slices with a small area. Following the previous approach, Figure 8 shows the graphical relationship between these values and a linear model.

As we can observe, most MRI slices below the 0.88 threshold (*Conf* − *Valid* − *IC* < 0.88) are slices with a small area compared to the rest of the slices (*Conf* − *Valid* − *IC* ≥ 0.88). Also, to avoid being too strict when discarding slices with an *Conf* − *Valid* − *IC* value close to 0.88, we establish a second threshold of 0.83, thus we use the range [0.83 ≤ *Conf* − *Valid* − *IC* ≤ 0.88] to define a second linear model. The resulting model is the red dashed line in Figure 8. This model is defined with a negative slope to compensate for an *Conf* − *Valid* − *IC* value close to 0.83 with a not-so-small area (and therefore fewer artifacts), and thus the slices with a small area that are selected will have an *Conf* − *Valid* − *IC* value close to or greater than 0.88 (an internal cavity with acceptable resolution).

The thresholds and the two linear models used to classify slices were manually selected based on visual inspection of the plotted distributions and expert judgment in collaboration with cardiologists. While this approach has proven effective for the current dataset, we recognize the importance of automating this process. In future work, we plan to approach this as a machine learning classification problem and explore the use of automatic thresholding techniques or classifiers such as decision trees, support vector machines, or ensemble models.

**Figure 9:**
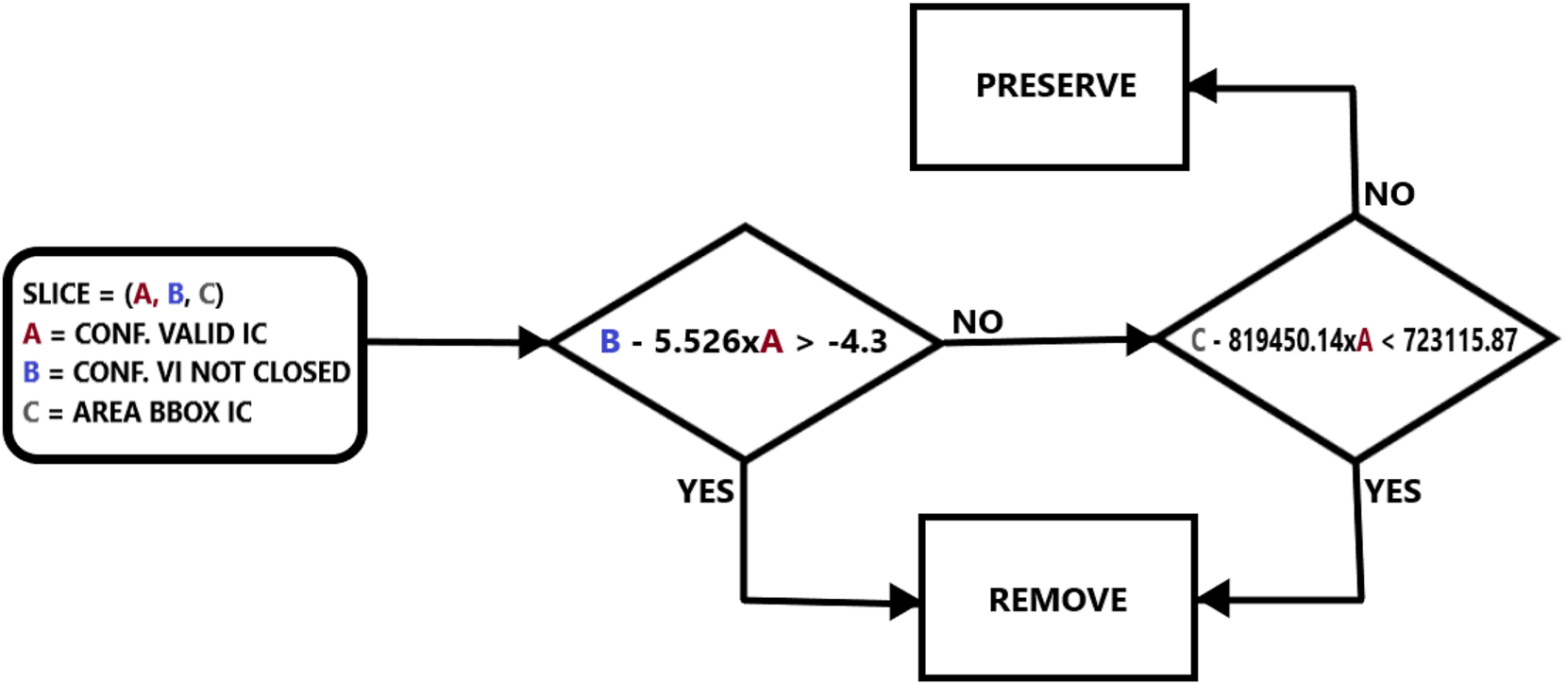
Flowchart establishing a criterion for automatically discarding slices from the dataset.

Finally, thanks to the two linear models we have obtained, we are able to design a flowchart (Figure 9) that describes the cardiology dataset cleaning method. Then, we can easily translate this flowchart into an algorithm that processes MRI slices as value triplets, as described earlier, and purges the dataset.

**Figure 10:**
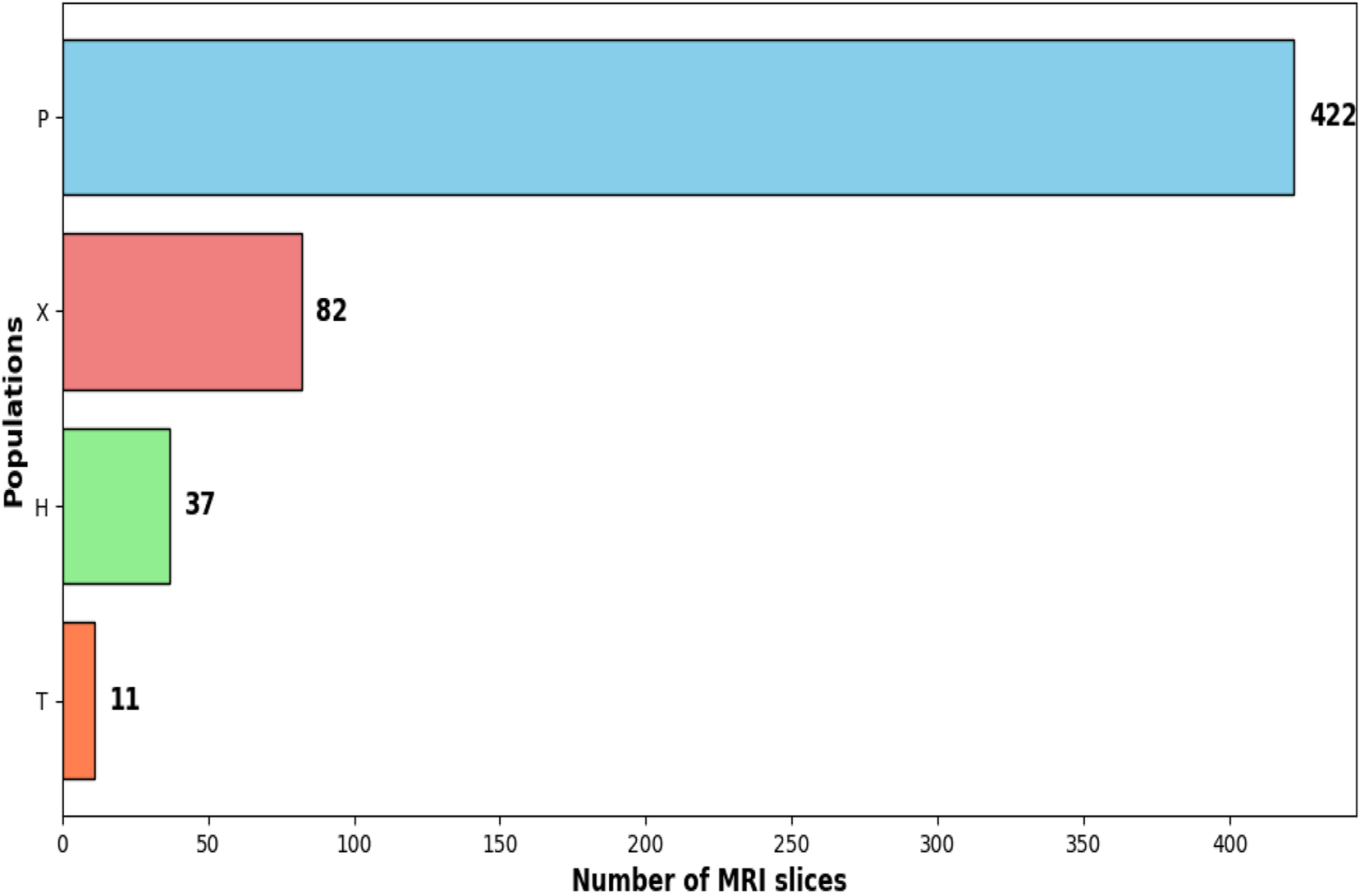
Number of MRI slices to be discarded for each patient group according to the flowchart in Figure 9.

The MRI slices to be discarded according to the flowchart amount to 552 (16 % of the total), leaving 2097 images in the purged dataset related to 449 patients, for whom the target *TV*% is readjusted if any slices were removed. Figure 10 shows how the 552 slices to be discarded are distributed among the different patient groups.

The justification and evaluation of this assumption will be provided in the results section (Section 3), where we analyze the impact of removing these problematic slices on the overall performance of the model.

### 2.4 Architecture ViTUNeT

**Figure 11:**
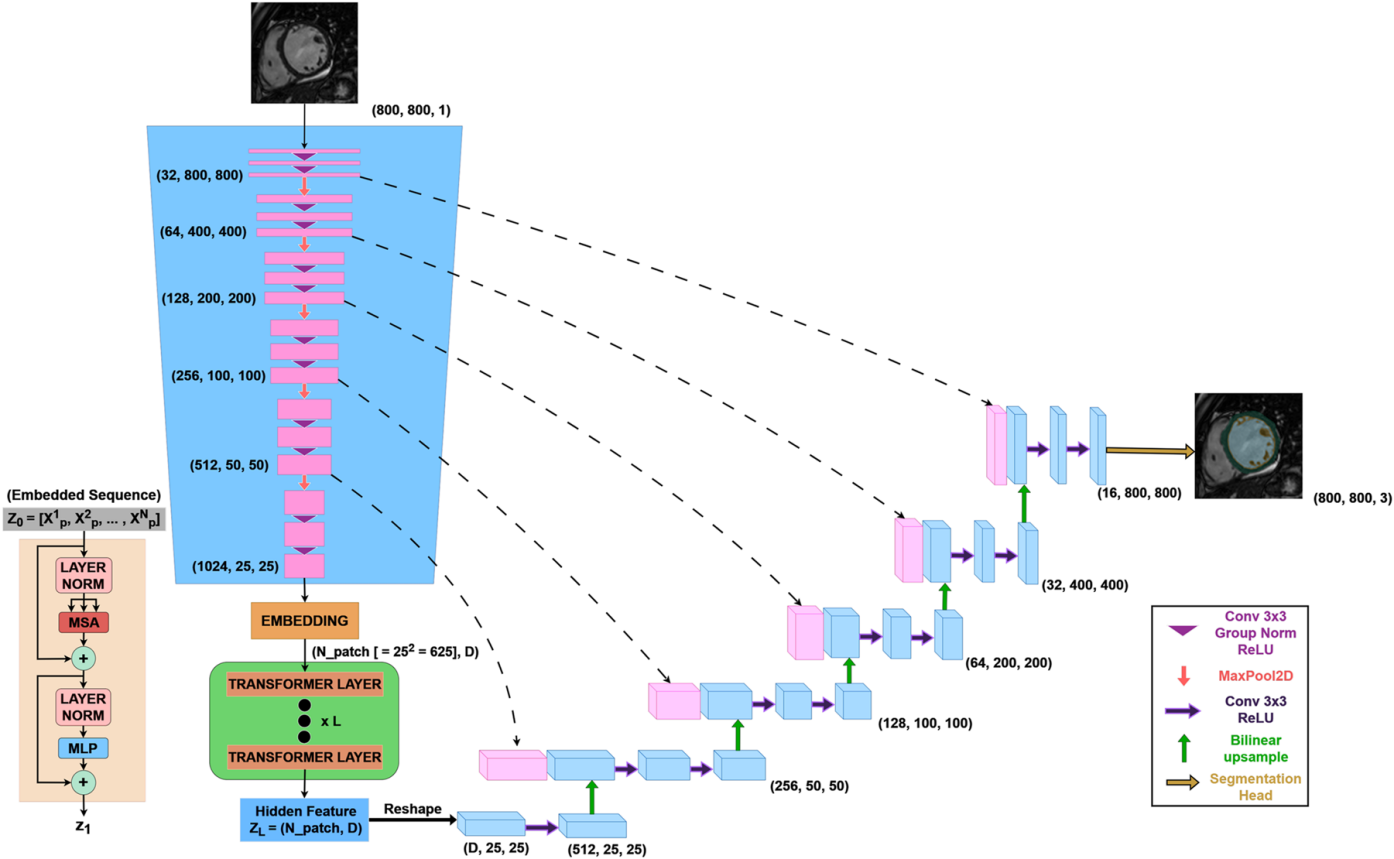
ViTUNeT architecture. Inspired by the TransUNet architecture [18].

This section introduces a convolutional neural network architecture named ViTUNeT, depicted schematically in Figure 11. This architecture was presented in our previous paper [22]. ViTUNeT is inspired by the TransUNet architecture [18].

In summary, ViTUNeT is designed to leverage the strengths of both Convolutional Neural Networks (CNNs) and Transformers, providing a robust framework for medical image segmentation.

The model follows an encoder-decoder architecture, where the encoder consists of a feature extractor based on U-Net and a Vision Transformer (ViT) module that enhances global contextual understanding.

**Convolutional Feature Extractor:** The convolutional encoder follows a hierarchical structure with multiple levels, each composed of convolutional blocks, batch normalization, and ReLU activations, followed by max-pooling layers. These operations progressively increase the number of feature maps while reducing their spatial resolution, allowing the model to extract multi-scale spatial features.

**Transformer-Based Global Processing:** Unlike traditional CNN-based architectures, ViTUNeT incorporates a Transformer encoder to capture long-range dependencies and global relationships within the images. The Transformer module receives a flattened representation of feature patches extracted from the CNN encoder and processes them using self-attention mechanisms. This enables the model to capture better structural patterns in cardiac MRI scans, essential for distinguishing trabeculated and compacted myocardial regions.

**Fusion and Decoding:** The features extracted by the Vision Transformer are fused with the hierarchical representations from the convolutional encoder, ensuring that the model retains fine-grained spatial details and global contextual information. This fusion facilitates an effective spatial recovery during the decoding phase, where a symmetrical CNN decoder progressively upsamples the feature maps to reconstruct the segmented output.

**Final Segmentation Output:** The network concludes with a convolutional segmentation head, which applies a softmax activation function to classify each pixel into one of four anatomical regions: the left ventricular internal cavity, the myocardial compact layer, the trabecular zone, and the background.

By combining local feature extraction through CNNs with global feature modeling through Transformers, ViTUNeT achieves a balanced trade-off between precision and robustness, significantly improving performance in complex left ventricle segmentation tasks.

#### 2.4.1 Combining YOLOv8 with the ViTUNeT model for LVNC

**Figure 12:**
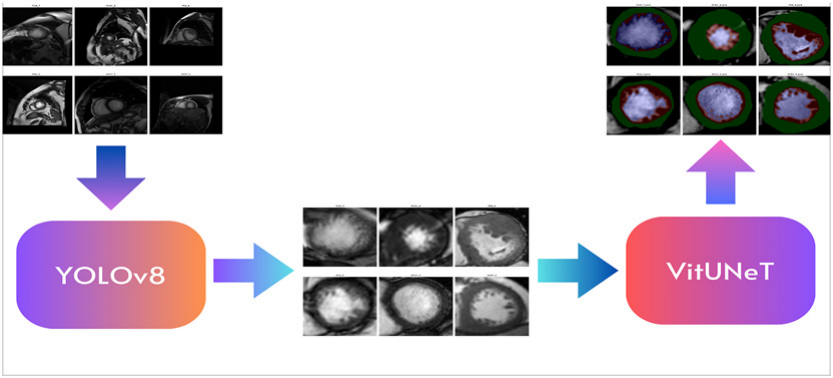
Integration of the YOLOv8 model with the ViTUNeT model.

We describe our method for integrating a YOLOv8 model, trained on our cardiology dataset to detect the left ventricle, with a model using the ViTUNeT architecture. Figure 12 provides a schematic representation of this integration process. We will refer to this method as YOLOv8+ViTUNet.

First, the YOLOv8 model processes short-axis MRI images to define a Region of Interest (ROI) by drawing a bounding box around the left ventricle, directing the convolutional segmentation model to focus on this area. To ensure that the generated ROI fully contains the left ventricle, we adjust the side length of the ROI using the probability with which the YOLO model detects a valid left ventricle, according to the following formula:

l_resize=max(w_roi,h_roi)×(1+(1−prob_YOLO))

where:

– *l*_*resize*: The adjusted side length of the final square ROI.
– *w*_*roi*, *h*_*roi*: The width and height of the original ROI, respectively.
– *prob*_*YOLO*: The probability assigned by the YOLO model for detecting the left ventricle.

Thus, this formula expands the ROI more significantly for slices where the YOLO model is more difficult to detect.

Next, each ROI is converted to a single grayscale channel and resized to 800 × 800 pixels to standardize input dimensions. Finally, Z-score normalization is applied to normalize the pixel intensity distribution. These preprocessing steps prepare the data for training the ViTUNeT model.

We aim to achieve the following benefits with this approach of using the YOLO model to guide the convolutional model in image segmentation: firstly, to prevent the convolutional model from mistakenly identifying other structures that resemble the LV in MRI slices; secondly, to ensure uniformity in the size of the LV across the MRI images of a patient, as the left ventricle may appear smaller in the extreme slices compared to those in the center of the series.

#### 2.4.2 Training method for ViTUNeT model

The proposed model is trained using images corresponding to unique 2D cardiac MRI slices with a resolution of 800 × 800 pixels. To normalize the values, a Z-score standardization is also applied to each original slice. During training, data augmentation is employed through random rotations of 90°, 180°, or 270° in the images with a probability of 0.25.

The loss function used is a linear combination of two components: the *Lovász-Softmax loss* [26] $\left(L_{L}\right)$ and the weighted *binary cross-entropy loss* $\left(L_{BC}\right)$ :

$$L=L_{L}+L_{BC}$$

This choice of loss function addresses the class imbalance issue, as the trabecular area is smaller than the other areas. The RAdam optimizer [27] minimizes the loss function, with an initial learning rate of 0.005.

**Figure 13:**
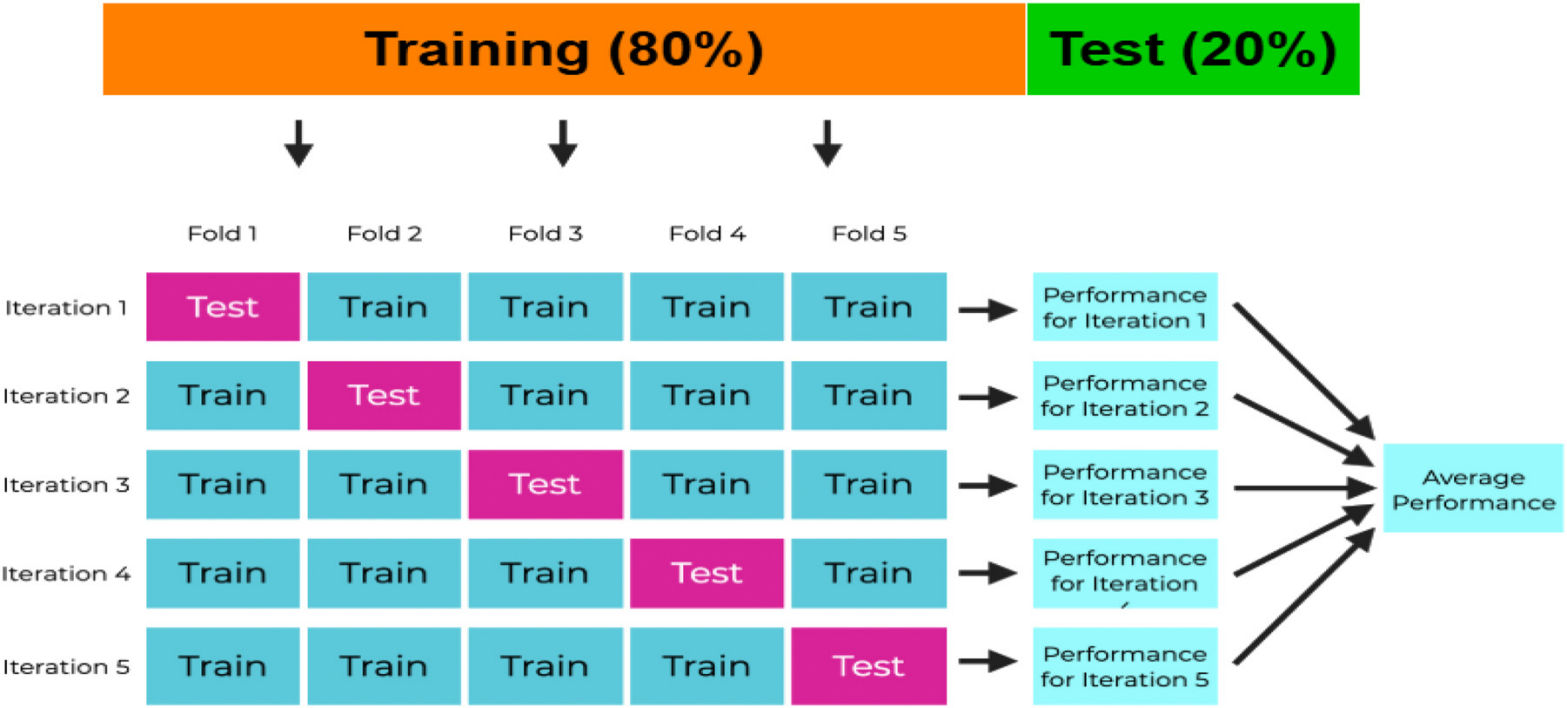
Generic scheme of our 5-fold cross-validation Process.

Additionally, to more robustly assess the segmentation quality of the models, a 5-fold stratified cross-validation process is employed during training. As described in Figure 13, the complete dataset is divided into 80 % of patients for training and 20 % for testing. Next, 5 folds are created within the training set, where each fold consists of 2 subsets: training (80 %) and validation (20 %). It is important to highlight that the division of the dataset is done “by patients,” not by slices, ensuring that a patient’s slices are not distributed across both the training and validation sets. Stratification is based on the 27.4 % threshold rule described in subsection 2.1. This ensures that the dataset is divided into five equally sized, non-overlapping folds and that the distribution of patients with LVNC and those without the disease is as balanced as possible across the different training and validation sets.

## 3 Results and discussion

In this section, we present the results obtained in our study for different approaches to developing a segmentation model. All the results are from inference, meaning they are obtained from a test set, which consists of 20 % of the MRI slices from the new dataset used in this study.

### 3.1 ViTUNet

First, we examine why it is necessary to retrain the ViTUNet model on the new dataset presented in subsection 2.2. Table 1 shows the inference accuracy for different patient groups in the new dataset, comparing the results obtained by the ViTUNet model trained on the old dataset from previous studies [22] and the model trained on the new dataset.

**Table 1:** Metrics for each patient group obtained in the inference by the ViTUNet model on the new dataset. The model was trained on the old dataset (Old) and the new dataset (New). EL is the external layer, IC is the inner cavity, T are the trabeculae, and the Average Dice Coefficient is the overall average Dice coefficient.

Population	Dice EL		Dice IC		Dice T		Average Dice	
	Old	New	Old	New	Old	New	Old	New
P	0.886	0.900	0.950	0.957	0.840	0.856	0.894	0.907
H	0.873	0.877	0.946	0.948	0.800	0.839	0.886	0.888
X	0.887	0.884	0.961	0.964	0.846	0.869	0.895	0.909
T	0.869	0.874	0.948	0.953	0.790	0.832	0.867	0.887

The patient groups where the model shows the lowest accuracy when trained on the old dataset are H and T. The size of group H has tripled compared to the previous version, and group T is new and was not used during training. Based on these results, we conclude that the new slices from these groups introduce properties that need to be learned, leading to the decision to retrain the model on the new dataset.

From Table 1, we observe that training the ViTUNet model on the new dataset improves the Dice coefficients for the different regions of interest, particularly the trabecular region, in patient groups H, T, and X. This increase in accuracy justifies the retraining of the ViTUNet model on the new dataset used in this study.

### 3.2 YOLOv8+ViTUNet

We use the term *dataset*_*yolo*_ to refer to the dataset of regions of interest around the LV. This dataset is obtained by applying the YOLOv8 model to the cardiology dataset with the MRI slices in their original state. Following this nomenclature, we use *new* − *dataset*_*yolo*_ to refer to the new dataset processed with YOLOv8.

Table 2 summarizes the inference accuracy of the ViTUNet model on *new* − *dataset*_*yolo*_.

**Table 2:** Metrics for each patient group obtained in the inference by ViTUNet model on the *new* − *dataset*_*yolo*_. EL is the external layer, IC is the inner cavity, T are the trabeculae, and the Average Dice Coefficient is the overall average Dice coefficient.

Population	Dice EL	Dice IC	Dice T	Average Dice
P	0.902	0.948	0.850	0.900
H	0.883	0.935	0.818	0.907
X	0.889	0.961	0.871	0.907
T	0.876	0.952	0.832	0.883

The Dice coefficients presented in this table show that the results are similar or slightly worse than those obtained in Table 1. This happens because, by setting an ROI around the LV and zooming in, the number of pixels corresponding to complex areas, such as trabeculae or artifacts, increases, and therefore, the segmentation model has a higher probability of making errors when classifying pixels.

### 3.3 Results on the cleaned dataset

In this section, we present the results of the different approaches for training and evaluating the new cardiology dataset after being cleaned using the method from Figure 9. The approaches compared are ViTUNet and YOLOv8+ViTUNet.

Table 3 presents the inference results for each approach, showing the Dice coefficients for different patient groups. The table includes results obtained using ViTUNet alone and those obtained with the YOLOv8+ViTUNet pipeline.

**Table 3:** Comparison of inference metrics for each patient group using ViTUNet and YOLOv8+ViTUNet on the cleaned dataset. EL: external layer, IC: inner cavity, T: trabeculae, Avg. Dice: overall average Dice coefficient.

Population	Dice EL		Dice IC		Dice T		Avg. Dice	
	ViTUNet	YOLOv8+ViTUNet	ViTUNet	YOLOv8+ViTUNet	ViTUNet	YOLOv8+ViTUNet	ViTUNet	YOLOv8+ViTUNet
P	0.908	0.912	0.961	0.958	0.870	0.863	0.913	0.911
H	0.898	0.883	0.964	0.950	0.857	0.840	0.907	0.892
X	0.892	0.893	0.968	0.966	0.873	0.872	0.911	0.910
T	0.882	0.878	0.973	0.971	0.925	0.920	0.927	0.924

From Table 3, we observe that both approaches yield similar Dice coefficients across different regions of interest. However, in some cases, ViTUNet alone achieves slightly higher accuracy, whereas in others, the YOLOv8+ViTUNet pipeline performs comparably or slightly lower.

These results suggest that, while the YOLOv8+ViTUNet approach does not significantly degrade performance, the additional processing step introduced by YOLOv8 does not lead to a substantial improvement in segmentation accuracy. Further analysis would be needed to assess whether this approach provides advantages in specific scenarios.

### 3.4 Comparison of results

Now, we compare the results of the different approaches regarding segmentation quality in the trabecular zone (the most relevant for our purposes).

**Figure 14:**
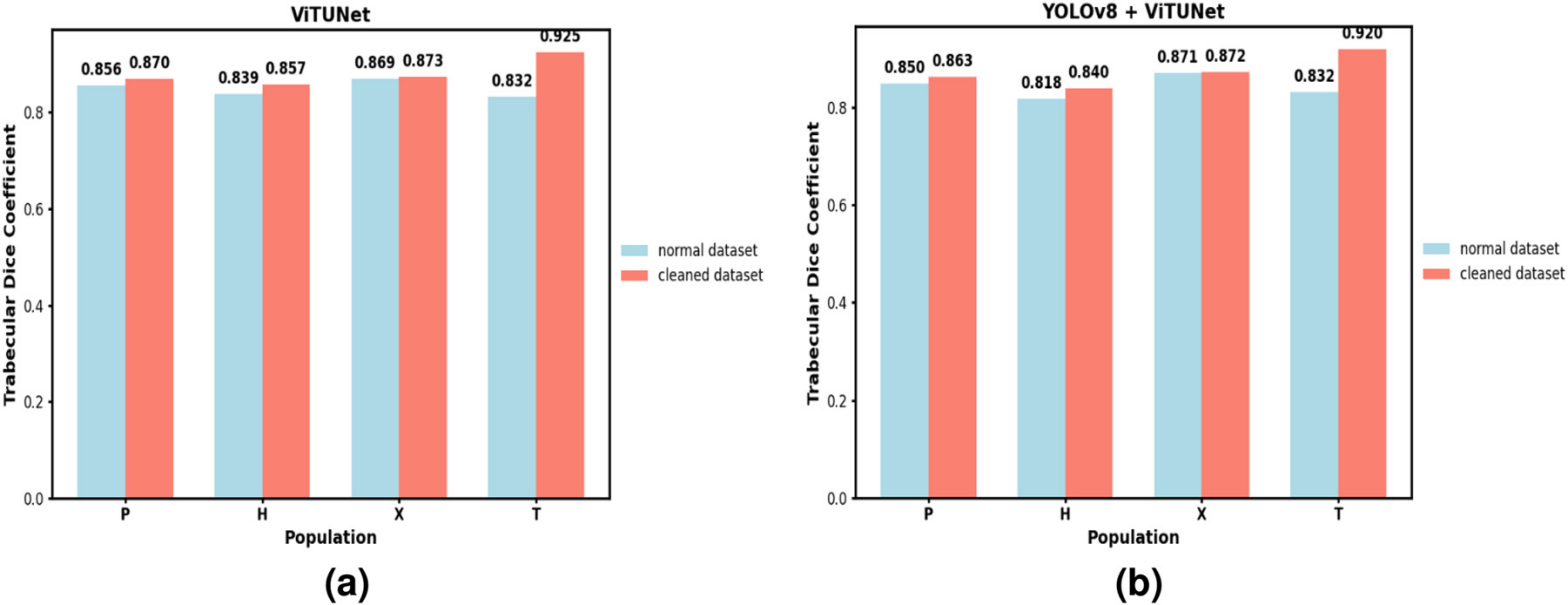
Comparative graphs of the Dice coefficient in the trabecular zone for different patient groups. Blue bars represent results using the normal dataset, while red bars correspond to the cleaned dataset. (a) ViTUNet model. (b) YOLOv8+ViTUNet approach.

Figure 14 presents the results of both the ViTUNet model and the YOLOv8+ViTUNet approach in a single comparative figure. Each subfigure shows the Dice coefficient across different patient groups, comparing results obtained with the normal dataset and the cleaned dataset.

As we can see in Figure 14, the best results are achieved when working with the cleaned dataset. This is expected, as our method for removing problematic slices discards images with small areas and artifacts that lack an internal ring-shaped cavity. Consequently, segmentation models trained on the cleaned dataset learn from slices with a more consistent morphology, improving performance.

**Figure 15:**
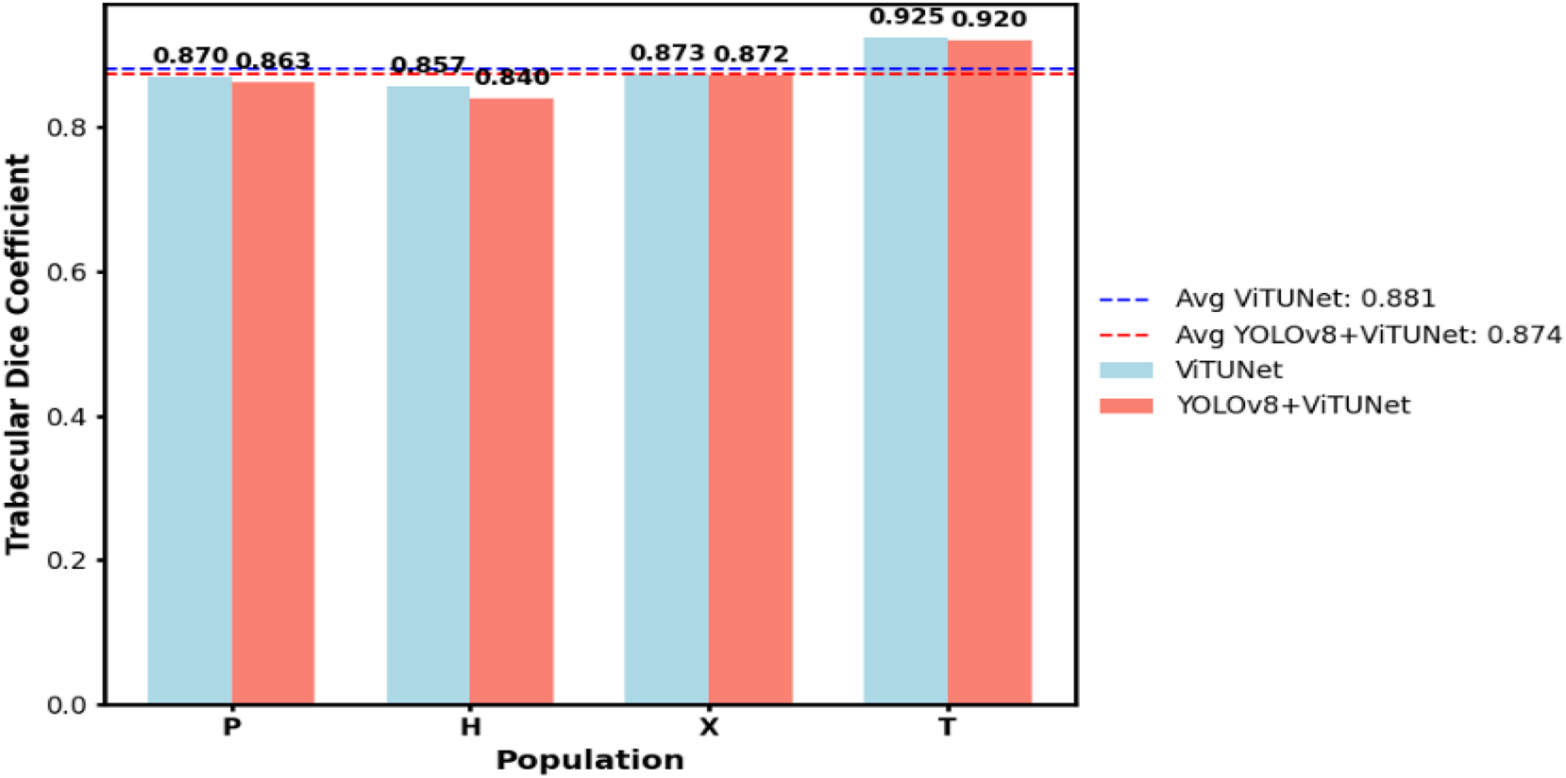
Comparative graph, in the cleaned dataset, of the Dice coefficients in the trabecular zone generated by the ViTUNet approach and the YOLOv8+ViTUNet approach.

Finally, in Figure 15, we present a graph to compare the ViTUNet and YOLOv8+ViTUNet approaches. This graph shows that in addition to the Trabecular Dice coefficients for each patient group, two horizontal lines indicate the average Trabecular Dice coefficient for each approach. We conclude that the ViTUNet model slightly outperforms the approach that uses a YOLOv8 model in the preprocessing phase.

The reason this occurs is that by converting each MRI slice into an ROI around the LV and resizing that ROI to 800 × 800 pixels (it’s like zooming in), the more controversial areas, such as the trabecular zone and the artifacts, occupy a more significant number of pixels, increasing the chance of segmentation errors. Additionally, we have noticed that images from group H tend to contain artifacts and have poorer resolution than those from the other groups. To address this limitation, future work could explore more sophisticated ROI preprocessing strategies, such as adaptive cropping based on trabecular density or context-aware padding to preserve anatomical context. Furthermore, incorporating attention-based refinement stages after segmentation might help the model focus more effectively on relevant structures while reducing the influence of misleading artifacts.

## 4 Conclusions and future work

This study presents ViTUNet, an architecture combining UNet and Vision Transformer, to segment the left ventricle in short-axis MRI scans. To enhance segmentation accuracy, we incorporate YOLOv8 to identify the left ventricle, allowing ViTUNet to focus on the correct region of interest and minimize confusion with similar structures.

With a more robust dataset than in previous studies, we assess the performance of ViTUNet with YOLOv8 across different patient groups and improve accuracy. However, we identify specific MRI slices in the dataset that present challenges, limiting our improvement despite using YOLO to focus on regions of interest around the ventricle. The main challenges include low resolution of slices, controversial trabecular areas, artifacts in extreme slices, imbalance between slices with few and many trabeculae, and some incorrect target segmentations.

Therefore, to address the inherent limitation of improvement in the cardiology dataset, we present a method based on two YOLOv8 models that automatically identify and remove problematic slices. By applying this method to the dataset, we observe a moderate improvement in accuracy. While data cleaning appears to contribute to better model performance, it is important to acknowledge that other factors, such as model architecture and hyperparameter tuning, may also play a role in the observed results.

Future work should focus on refining the method for distinguishing between problematic and non-problematic slices, for example, by testing other classification models such as Random Forest or Support Vector Machine. To this end, we should label many problematic slices and enhance the YOLOv8 models. Additionally, it would be interesting to explore image processing techniques that mitigate artifacts in MRI slices. It is also essential to identify and reprocess those MRI slices for which the target segmentation mask was not obtained correctly in the past. Adding patients with few trabeculae could improve results since most slices in the dataset exhibit trabecular tissue in a ring shape around the internal cavity, causing the models to learn this pattern and potentially hallucinate in slices that do not present such a pattern.
